# Supplementary material for: ROCK activity regulates functional tight junction assembly during blastocyst formation in porcine parthenogenetic embryos
Source: PeerJ. 2016 Apr 11;4:e1914. doi: 10.7717/peerj.1914 (PMC4830244; doi:10.7717/peerj.1914)
Supplement: Supplemental Information 1 — The blastocyst development rates of the four-cell stage embryos exposed to different concentrations of Y-27632 (control 0, treatment 10, 20, and 100 µM). [file peerj-04-1914-s001.pdf]

	embryo	blastocyst	%
control	27	12	0.444444
10	22	6	0.272727
20	24	4	0.166667
100	25	0	0

control	embryo	blastocyst	%
10	26	16	0.615385
20	24	4	0.166667
100	25	2	0.08
	22	0	0

control	embryo	blastocyst	%		mean	s.e.m
10	24	14	0.583333		0.547721	0.052461
20	24	4	0.166667		0.20202	0.035354
100	25	2	0.08		0.108889	0.028889
	23	0	0		0	0
