# Supplementary material for: ROCK activity regulates functional tight junction assembly during blastocyst formation in porcine parthenogenetic embryos
Source: PeerJ. 2016 Apr 11;4:e1914. doi: 10.7717/peerj.1914 (PMC4830244; doi:10.7717/peerj.1914)
Supplement: Supplemental Information 2 — The fractions of embryos treated with 20 µM Y-27632 at the four-cell or morula stages that developed to the morula and blastocyst stages, respectively, at 96 and 120 h.p.a. [file peerj-04-1914-s002.pdf]

				96h			120
	4-cell	<8cell	Morula	Blastocyst	<8cell	Morula	Blastocyst
Control	92		56			29	36
		0.391304348	0.608696	0	0.293478	0.315217	0.391304
Y-27632	92		14	0		14	0
		0.847826087	0.152174	0	0.847826	0.152174	0

	96	120
	morula	blastocyst
Control	71	36
	0.49296	0.507042254
Y-27632	79	7
	0.91139	0.088607595
