# Supplementary material for: ROCK activity regulates functional tight junction assembly during blastocyst formation in porcine parthenogenetic embryos
Source: PeerJ. 2016 Apr 11;4:e1914. doi: 10.7717/peerj.1914 (PMC4830244; doi:10.7717/peerj.1914)
Supplement: Supplemental Information 3 — Total cell number of embryos at 120 h.p.a after the treatment of morula embryos with 20 µM Y-27632. [file peerj-04-1914-s003.pdf]

	Control	20 µM Y-27632
	30	19
	24	13
	32	21
	45	18
	17	17
	21	13
	35	19
	39	17
	18	17
	21	11
	32	17
	45	13
	21	17
	20	13
	32	15
	39	18
	18	13
	25	22
	17	17
	44	18
	33	19
	29	13
	32	12
	45	18
mean	29.75	16.25
s.e.m	1.95627	0.608663
