# Supplementary material for: ROCK activity regulates functional tight junction assembly during blastocyst formation in porcine parthenogenetic embryos
Source: PeerJ. 2016 Apr 11;4:e1914. doi: 10.7717/peerj.1914 (PMC4830244; doi:10.7717/peerj.1914)
Supplement: Supplemental Information 4 — Relative levels of CXADR, OCLN, TJP1, and CDH1 transcripts in embryos treated with 10 µM Y-27632 in comparison to their levels in non-treated, control blastocysts. [file peerj-04-1914-s004.pdf]

	CXADR	OCLN	TJP1	CDH1
Rocki	0.408	0.259	0.558	0.255
Rocki	0.457	0.235	0.518	0.215
Rocki	0.273	0.315	0.415	0.305
Rocki	0.215	0.452	0.408	0.288
Rocki	0.247	0.315	0.552	0.156
Rocki	0.153	0.335	0.498	0.158
Rocki	0.148	0.452	0.604	0.155
Rocki	0.225	0.298	0.507	0.255
mean	0.266	0.333	0.508	0.223
s.e.m	0.046	0.033	0.028	0.025
